# Genome-Wide Identification of Flowering-Time Genes in *Brassica* Species and Reveals a Correlation between Selective Pressure and Expression Patterns of Vernalization-Pathway Genes in *Brassica napus*

**DOI:** 10.3390/ijms19113632

**Published:** 2018-11-18

**Authors:** Haojie Li, Yonghai Fan, Jingyin Yu, Liang Chai, Jingfang Zhang, Jun Jiang, Cheng Cui, Benchuan Zheng, Liangcai Jiang, Kun Lu

**Affiliations:** 1 Crop Research Institute, Sichuan Academy of Agricultural Science, Chengdu 610066, China; lhjie16@163.com (H.L.); chailiang1982@126.com (L.C.); zhangjinfang567@163.com (J.Z.); jiangjun227@163.com (J.J.); ChengCui@163.com (C.C.); zhengbenchuan1987@163.com (B.Z.); 2 College of Agronomy and Biotechnology, Southwest University, Beibei, Chongqing 400715, China; fyh1212@email.swu.edu.cn; 3 Academy of Agricultural Sciences, Southwest University, Beibei, Chongqing 400715, China; 4 Key Laboratory of Biology and Genetic Improvement of Oil Crops, Ministry of Agriculture; Oil Crops Research Institute, the Chinese Academy of Agricultural Sciences, Wuhan 430062, China; yujingyin@caas.cn

**Keywords:** *Brassica napus*, evolutionary selection pressure, expression pattern, flowering time

## Abstract

Flowering time is a key agronomic trait, directly influencing crop yield and quality. Many flowering-time genes have been identified and characterized in the model plant *Arabidopsis thaliana*; however, these genes remain uncharacterized in many agronomically important *Brassica* crops. In this study, we identified 1064, 510, and 524 putative orthologs of *A.* *thaliana* flowering-time genes from *Brassica napus*, *Brassica rapa,* and *Brassica oleracea*, respectively, and found that genes involved in the aging and ambient temperature pathways were fewer than those in other flowering pathways. Flowering-time genes were distributed mostly on chromosome C03 in *B. napus* and *B. oleracea*, and on chromosome A09 in *B. rapa*. Calculation of non-synonymous (*Ka*)/synonymous substitution (*Ks*) ratios suggested that flowering-time genes in vernalization pathways experienced higher selection pressure than those in other pathways. Expression analysis showed that most vernalization-pathway genes were expressed in flowering organs. Approximately 40% of these genes were highly expressed in the anther, whereas flowering-time integrator genes were expressed in a highly organ-specific manner. Evolutionary selection pressures were negatively correlated with the breadth and expression levels of vernalization-pathway genes. These findings provide an integrated framework of flowering-time genes in these three *Brassica* crops and provide a foundation for deciphering the relationship between gene expression patterns and their evolutionary selection pressures in *Brassica napus*.

## 1. Introduction

The phase transition from vegetative to reproductive growth is a crucial step in the plant’s life cycle, especially in relation to plant adaptation and crop productivity [1,2,3,4]. Generally, flowering time is central to reproductive success and isolation [1,3]. The process of flowering involves all aspects of plant growth, including cell and organ differentiation, cell division, and gene expression, which are controlled by both genetic and environmental factors. Most plants restrict flowering to a specific time of year when conditions are optimal for pollination and seed development [5]. To achieve this, plants depend on reliable environmental factors, such as temperature, day length, nutrient and water availability [5].

Flowering time involves a variety of genes and is regulated by internal signals [6,7]. Environmental factors play additional key roles in stimulating internal regulatory factors [8,9]. Switching the shoot apical meristem (SAM) from vegetative to reproductive growth is controlled by both environmental and endogenous signals, and this process is affected mainly by photoperiodic promotion, autonomous enhancement, flowering inhibition, vernalization (the acceleration of flowering by low temperatures and the suppression of specific genes), and gibberellin induction [10]. These factors act on the inflorescence/floral meristem in combination, resulting in the conversion of the apical meristem to the inflorescence/floral meristem. The inflorescence/floral meristem then forms the floral organs by activating the expression of floral organ-specific genes. 

The molecular mechanisms underlying flowering have been extensively studied in the model plant *A. thaliana*, and these are summarized in the FLOR-ID (Flowering Interactive Database), which is an interactive database of flowering-time gene networks [11]. The *A. thaliana* flowering-time networks comprise a total of 306 genes, and most of these are members of multigene families. According to the FLOR-ID, flowering-time genes are allocated into seven pathways: photoperiodic/lighting and signaling, vernalization, ambient temperature, aging, sugar, hormones, and autonomous. Genes involved in multiple pathways are regarded as “flowering-time integrators” [11].

Photoperiodic/lighting and signaling pathways consist mainly of three groups of genes: photoreceptor, circadian clock components, and photoperiodic pathway genes. Plant light perception is accomplished primarily by five known classes of photoreceptors: *UVB-RESISTANCE* 8 (*UVR8*); the blue light receptor, *ZEITLUPE* (*ZTL*), *cryptochrome* (*CRY*) and *phototropin* (*PHOT*); and the red/far-red light receptor, *phytochrome* (*PHY*) [5] Plant circadian clock was entrained primarily through the sensing of light and temperature, allows plants to regulate metabolic pathways and measure the variations in day length, inducing flowering under specific induced daylength conditions [12,13,14]. For example, two *A. thaliana MYB* transcription factors, *CIRCADIAN CLOCK ASSOCIATED 1* (*CCA1*) and *LATE ELONGATED HYPOCOTYL* (*LHY*) are expressed mainly in the morning, while *PSEUDO-RESPONSE REGULATOR 1* (*PRR1*, also called *TIMING OF CAB EXPRESSION 1*, *TOC1*) is expressed primarily at night. These three genes form the central oscillator feedback loop of the circadian clock [5]. To date, the most studied genes in the photoperiodic pathway are *CONSTANS* (*CO*) and *FLOWERING LOCUS T* (*FT*). *CO* encodes a zinc finger transcription factor that plays a crucial role in regulating flowering time in response to photoperiodic signals [15]. Prolonged sunlight leads to the accumulation of *CO*, and directly activates the expression of the flowering pathway integrators *SUPPRESSOR OF OVEREXPRESSION CO1* (*SCO1*) and *FT* [16]. *FLOWERING LOCUS C* (*FLC*) is a transcription repressor which negatively regulates the expression of *FT* interacting with other factors from the vernalization pathway [17,18]. *FLC* encodes a transcription factor with a MADS domain, and functions together with *FRIGIDA* (*FRI*) to control the late-flowering phenotype in *A. thaliana*. Moreover, *FRI* promotes the expression of *FLC*, leading to delayed flowering [19]. Vernalization inhibits the function of *FRI,* thus maintaining low expression levels of *FLC*, promoting flowering in the second year [20]. In addition, ambient temperature, age, sugar, hormones, and autonomous pathway genes play key roles in modulating the flowering response [17,18].

Understanding the control of flowering time in crops is not only important for improving crop yield, but also provides a theoretical basis for cultivating crops using diverse farming practices. To date, flowering-time genes have been extensively identified and studied in several crops, including cotton (*Gossypium raimondii*), radish (*Raphanus sativus*), wheat (*Triticum aestivum*), and barley (*Hordeum vulgare*) [21,22,23,24,25]. However, this is not the case for crops from the genus *Brassica*. *Brassica* is one of the most important genera of cruciferous plants, including many important vegetables, oil, and forage crops. Although several flowering-time genes have been characterized in *Brassica* species [26,27,28,29], their systematic investigation at the genome-wide level remains to be performed, especially in the important oilseed crop *B. napus*.

Here, we identified flowering-time genes in three *Brassica* species, *B. napus* and its two progenitors, *B. rapa* and *B. oleracea*, using 295 protein-coding flowering genes from *A. thaliana* as query sequences. We then compared the properties of these genes and gene family expansion status in different flowering-time pathways among the three species, which revealed a negative correlation between selective pressures and expression levels. We also assessed the correlation between selective pressures and expression breadth (i.e., the number of organs in which the genes are expressed) of vernalization-pathway genes. Studies of putative orthologs and of the relationship between selective pressures and expression patterns provided further information on the control of flowering time in *Brassica* species.

## 2. Results

### 2.1. Identification of Flowering-Time Genes in B. rapa, B. oleracea, and B. napus

A total of 306 genes involved in flowering were detected in *A. thaliana* from FLOR-ID [11], including 295 coding genes and 11 noncoding genes (Table S1). The results from the PfamScan analysis of the 295 coding-protein sequences showed that 276 members contained 117 different functional domains, such as 2OG-FeII_Oxy, AP2, HLH, and SRF-TF (Table S2). No functional domain existed in the remaining 19 members (Table S2).

For those domain-containing genes, the results derived from HMMER and BLASTP searches were intersected and identified a total of 985, 475, and 482 domain-containing flowering-time genes in *B. napus*, *B. rapa*, and *B. oleracea*, respectively (Tables S3–S5). For those non-domain-containing genes, the reciprocal BLASTP analysis was used and 79, 35, and 42 flowering-time genes were identified in *B. napus*, *B. rapa*, and *B. oleracea*, respectively (Tables S3–S5).

Using 295 *A. thaliana* flowering-time genes as queries, we identified a total of 1064, 510, and 524 flowering-time genes in *B. napus*, *B. rapa*, and *B. oleracea*, respectively (Table 1, Tables S3–S5). No orthologous genes were found corresponding to 20, 22, and 28 *A. thaliana* flowering-time genes in *B. napus*, *B. rapa*, and *B. oleracea*, respectively (Table S6). This includes 17 *A. thaliana* genes that were simultaneously lost in the three *Brassica* species, for example, *SNZ*, *ATJ3*, and *CIB5*. The identity of sequences between genes in *A. thaliana* and their corresponding orthologs ranged from 50% to 100%, with an average of 94.83%, 95.14%, and 94.89% to those in *B. napus*, *B. rapa*, and *B. oleracea*, respectively (Tables S3–S5).

### 2.2. Partition of Flowering-Time Genes to Different Pathways

Both *A. thaliana* and *Brassica* belong to the Brassicaceae family and share a high level of similarity among orthologous genes. According to the classification rule of flowering-time genes in *A. thaliana* [11], the *Brassica* flowering-time genes are classified into seven pathways: photoperiodic/lighting and signaling, vernalization, aging, ambient temperature, hormone, sugar and autonomous (Table 1). Interestingly, 34 genes in *A. thaliana* are involved simultaneously in two pathways, including photoperiodic/lighting and signaling and aging pathways, ambient temperature and autonomous pathways, ambient temperature and photoperiodic/lighting and signaling pathways, ambient temperature and vernalization pathways, photoperiodic/lighting and signaling and autonomous pathways, and vernalization and autonomous pathways (Table 1). For example, *TARGET OF EARLY ACTIVATION TAGGED* (*TOE*) genes are classified into photoperiodic/lighting and signaling and aging pathways. Moreover, more genes were involved in both photoperiodic/lighting and signaling and autonomous pathways than in other pathways, including 50 members from *B. napus*, 24 members from *B. rapa,* and 26 members from *B. oleracea* belonging to both these two pathways (Table 1). The ambient temperature pathway involved the fewest genes.

The photoperiodic/lighting and signaling pathway include the circadian clock, photoperiodism/light perception, and signaling pathways, which play crucial roles in regulating flowering time. In this study, 390, 187, and 193 members were identified in *B. napus*, *B. rapa,* and *B. oleracea*, respectively, as being involved in the photoperiodic/lighting and signaling pathway (Table 1, Tables S3–S5). In the circadian clock pathway, most of the flowering-time genes in *A. thaliana* possessed two to eight orthologs in *B. napus* and one to four orthologs in *B. rapa* and *B. oleracea*, such as *CCA1*, *CASEIN KINASE II BETA SUBUNIT 3* (*CKB3*), *EARLY FLOWERING 4* (*ELF4*), and *FIONA 1* (*FIO1*), but no orthologs were found for *LIGHT-REGULATED WD 2* (*LWD2*) and *ZTL* among the three *Brassica* species. Similarly, no orthologs were detected for *CRYPTOCHROME-INTERACTING BASIC-HELIX-LOOP-HELIX 5* (*CIB5*), *FLOWERING BHLH 2* (*FBH2*), *FLAVIN-BINDING-KELCH REPEAT-F BOX 1* (*ADAGIO3/ADO3*), *PHYTOCHROME D* (*PHYD*), *CALCIUM-DEPENDENT PROTEIN KINASE 33* (*CPK33*), *NUCLEAR FACTOR Y-SUBUNIT C3* (*NF-YC3*), and *SCHNARCHZAPFEN* (*SNZ*) in the photoperiodism/light perception and signaling pathways (Table S6). The key regulator in flowering time is *CO*, which integrates the information between the internal circadian clock and the external day-night cycles [30]. In this study, we identified two *CO* members in *B. napus* (*BnaA10g18430D* and *BnaC09g41990D*) and one copy in both *B. rapa* (*Bra008669*) and *B. oleracea* (*Bol030488*). In addition, nine, five, and five *CONSTANS-LIKE* (*COL*) genes were identified in *B. napus*, *B. rapa,* and *B. oleracea*, respectively. Three F-box family genes, putative orthologs of *Arabidopsis LOV KELCH PROTEIN 2* (*LKP2)*, *ZTL* and *FLAVIN-BINDING-KELCH REPEAT-F BOX 1* (*FKF1*); *LKP2* existed as six tightly linked copies in *B. napus* and three copies in *B. rapa* and *B. oleracea*, but *ZTL* and *FKF1* were both lost in these *Brassica* species.

The flowering-time integrator pathway consists of a small number of central floral pathway factors, which integrate signals from several pathways during floral transition [31], including the meristem response and development pathway. In this pathway, 72, 34, and 33 ‘integrator’ genes were identified from *B. napus*, *B. rapa,* and *B. oleracea*, respectively, using the 16 ‘integrator’ genes from *A. thaliana* (Table 1, Tables S3–S5). Previous studies have revealed that the expression of *FT* is a crucial step in flowering [17,18]. In this study, three copies of *FT* were found in *B. napus*, and one copy was identified in *B. rapa* and *B. oleracea*, respectively (Tables S3–S5). Other important flowering genes, such as *FLOWERING LOCUS F* (*FLF*), *LEAFY* (*LFY*), *SUPPRESSOR OF OVEREXPRESSION OF CO 1* (*COS1*), *SHORT VEGETATIVE PHASE* (*SVP*), and *TERMINAL FLOWER1* (*TFL*) were also detected in the three species, but no ortholog was found for either *SQUAMOSA PROMOTER BINDING PROTEIN-LIKE 4* (*SPL4*) or *XAANTAL2* (*XAL2*) in *B. oleracea* (Table S6).

Most plants that bloom in spring need to experience a cold period to overcome a block on flowering, which occurs through the process known as vernalization. In the vernalization pathway, 117, 61, and 57 flowering-time genes were identified in *B. napus*, *B. rapa,* and *B. oleracea*, respectively (Table 1, Tables S3–S5). Natural variation studies in *A. thaliana* illustrate that vernalization is strongly influenced by two dominant genes, *FLC* and *FRI*. *FLC* functions as the major repressor of flowering in the vernalization pathway and encodes a MADS-box DNA binding protein [20], and *FRI* encodes a nuclear protein that interacts with mRNA to regulate the level of *FLC* mRNA [23]. In this study, nine, three, and one copies of the *FLC* gene were identified in *B. napus*, *B. rapa,* and *B. oleracea*, respectively, with a striking expansion of *FLC* in *B. napus*. Whereas one copy of *FRI* was identified in both *B. napus* and *B. rapa*, no ortholog was found in *B. oleracea*.

### 2.3. Chromosomal Location Analysis

*B. napus* was formed by natural hybridization between two progenitors, *B. rapa* and *B. oleracea*, approximately 7500–10,000 years ago [32,33]. The A subgenome in *B. napus* is derived from the *B. rapa* genome while the C subgenome is derived from the *B. oleracea* genome. To determine the chromosomal location of flowering-time genes, all the genes were mapped onto the chromosomes of *B. napus*, *B. rapa*, and *B. oleracea*. A total of 870, 505, and 415 genes mapped to chromosomes, respectively (Figures S1–S3), and the remaining genes were assigned to unanchored scaffolds (Table S7). All chromosomes contained at least 30 genes. 

In *B. napus*, the highest number of genes was detected on chromosome C03, which contained 90 members. Seventy genes were detected on chromosome C04, and the lowest number of genes was found on chromosome A08, with only 31 members (Figure S1, Table S7). In *B. rapa*, the highest number of genes was detected on chromosome A09, which contained 71 members. Seventy genes were detected on chromosome A03, and the lowest number of genes was found on chromosome A10, which possessed 30 members (Figure S2, Table S7). In *B. oleracea*, chromosome C03 contained the highest number of genes, 75, followed by chromosome C04 with 54 members, and the lowest number of genes was 31 on chromosome C05 (Figure S3, Table S7).

A comparison of chromosome locations of flowering-time genes among *B. napus*, *B. rapa,* and *B. oleracea* showed that a total of 511 genes were localized on the A subgenome in *B. napus*, which is almost equal to the number of its orthologs in *B. rapa* (510 genes were identified in this study). However, a total of 550 genes were localized on the C subgenome in *B. napus*, which is much higher than the number of its orthologs in *B. oleracea* (524 genes were identified in this study), illustrating that flowering-time genes in the C subgenome underwent greater expansion than in the A subgenome in *B. napus*.

### 2.4. Gene Expansion Analysis

Previous studies predict that approximately 101,040, 41,100, and 45,500 protein-coding genes exist in the *B. napus* [32], *B. rapa* [34], and *B. oleracea* [35] genomes, respectively. A comparison between these three genomes and that of *A. thaliana* showed that the number of genes in *B. napus*, *B. rapa,* and *B. oleracea* was 4.04, 1.64, and 1.82 times that of *A. thaliana* at the whole-genome level, respectively, which was different to the number of flowering-time genes predicted in this study (3.61, 1.72, and 1.78 times that of *A. thaliana*, respectively) (Table 1), demonstrating that flowering-time genes expanded in *B. rapa*, but were lost in *B. napus* and *B. oleracea*.

The gene expansion ratios among the different flowering-time pathways were also uneven in the *Brassica* species studied here. The greatest expansion was detected in the flowering-time integrator pathway, with ratios of 4.50, 2.13, and 2.06 in *B. napus*, *B. rapa*, and *B. oleracea*, respectively (Table 1), which was higher than the average expansion at the whole-genome level, implying the importance of this pathway in the flowering of *Brassica* species. The minimum expansion ratio was in the ambient temperature pathway, with ratios of only 2.70, 1.30, and 1.20 in *B. napus*, *B. rapa,* and *B. oleracea*, respectively (Table 1).

**Figure 1:**
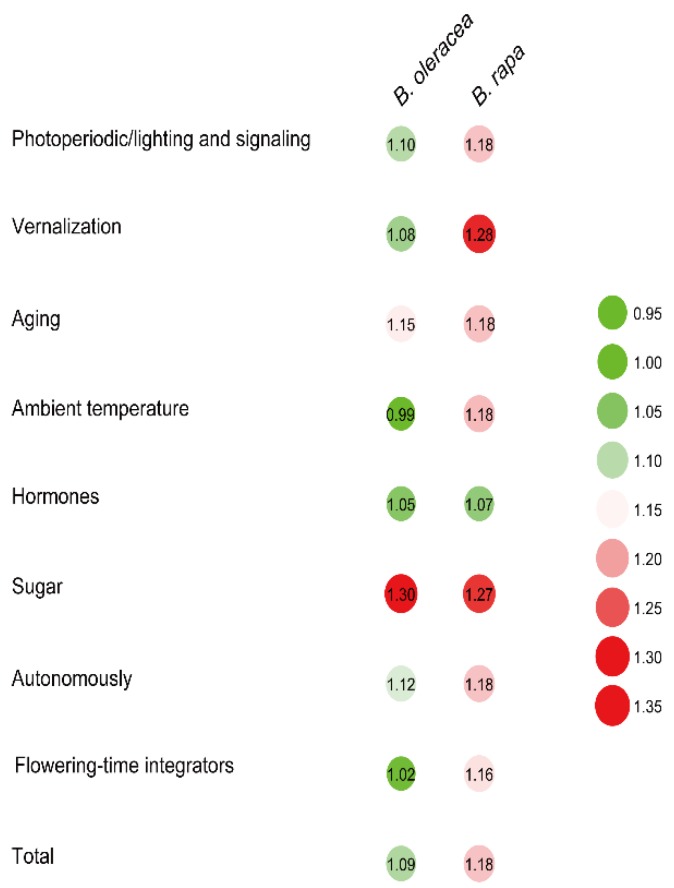
Gene expansion comparison among *B. napus*, *B. rapa*, and *B. oleracea*. The method is explained in Material and Methods and the colors represent the various degrees of gene expansion. If *Γ* is less than one in pathway *A*, the flowering-time pathway *A* in *B. napus* experienced an expansion event, while if greater than one, a contraction event occurred in this pathway. The *Γ* is greater, the contraction is more serious.

To accurately estimate the expansion status in the three *Brassica* species, the gene number ratio (*Γ*) was used in this study (see Material and Methods). Different *Γ* existed in different species for the same pathway. The *Γ* was 1.18 in *B. rapa* and 1.09 in *B. oleracea* for all flowering-time genes (Figure 1), illustrating that flowering-time genes in both *B. rapa* and *B. oleracea* showed greater expansion than in *B. napus*. Among the flowering time pathways, the *Γ* ranged from 1.07 (hormone pathway) to 1.28 (vernalization pathway) in *B. rapa*, and from 0.99 (ambient temperature pathway) to 1.30 (sugar pathway) in *B. oleracea* (Figure 1). The *Γ*s of all the flowering pathways in *B. rapa* and *B. oleracea* were higher than 1, except for the ambient temperature pathway in *B. oleracea*. In addition, the *Γ* in *B. rapa* was more than in *B. oleracea* in most pathways, except the hormone and sugar pathways. In general, flowering-time genes in *B. rapa* showed stronger expansion, followed by *B. oleracea* (Figure 1), demonstrating that more flowering-time genes were retained in the two progenitors than in *B. napus*.

### 2.5. Phylogenetic Analysis of Two Flowering-Time Gene Families

Plant phosphatidylethanolamine-binding protein (PEBP) family proteins share a PBP domain (PF01161.19) and consist of three subfamilies: FT, TERMINAL FLOWER 1 (TFL1), and MOTHER OF FT (MFT). FT induces flowering, TFL1 suppresses flowering, and MFT primarily regulates seed germination [36,37]. In *A. thaliana*, the PEBP family contains five flowering-time proteins: FT, TFL1, CENTRORADIALIS (ATC), BROTHER OF FT AND TFL1 (BFT), and TWIN SISTER OF FT (TSF). In this study, 21, 10, and 11 PEBP members were identified in *B. napus*, *B. rapa*, and *B. oleracea*, respectively (Tables S3–S5), and each *A. thaliana* PEBP protein possessed a few orthologs in the *Brassica* species. In addition, 18 MADS-box (a highly conserved N-terminal DNA binding domain) proteins in *A. thaliana* are involved in flowering time and contain the SRF-TF/K-box domain (PF00319.17/PF01486.16), such as AP1 (AGL7), FLC, MAF1-MAF5, and SVP (AGL22). We identified 79, 37, and 30 SRF-TF/K-box proteins in *B. napus*, *B. rapa*, and *B. oleracea*, respectively (Tables S3–S5). Most of the MADS-box proteins in *A. thaliana* possessed orthologs in the *Brassica* species, but no homologs were detected for FLM (MAF1/AGL27) and MAF5 (AGL68). In addition, XAL2 (AGL14), MAF3 (AGL70), and MAF4 (AGL69) were not detected in *B. oleracea*, and MAF2 (AGL31) was lost in both *B. napus* and *B. oleracea* (Table S6).

**Figure 2:**
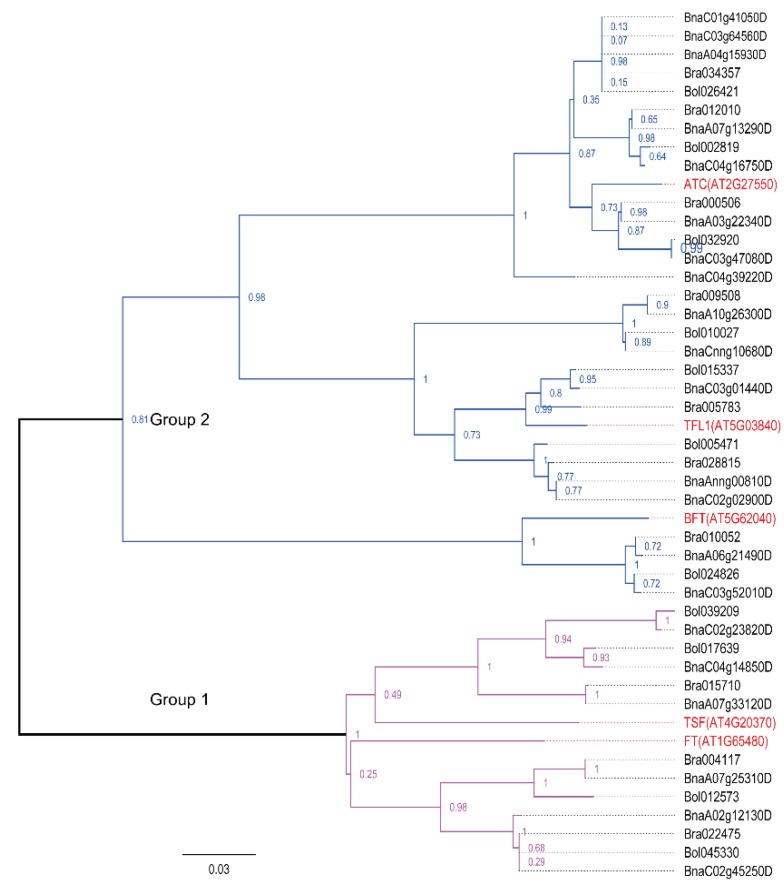
Phylogeny of PEBP family proteins in *A. thaliana*, *B. napus*, *B. rapa*, and *B. oleracea*. The phylogenetic tree was constructed using MEGA 7.0 with the neighbor-joining method (1000 bootstrap replicates) and displayed using FigTree v1.4.0. PEBP proteins involved in flowering are clustered into two distinct groups (Group 1, the purple clade; Group 2, the blue clade). The scale bar denotes the number of nucleotide replacements per site. PEBP: phosphatidylethanolamine-binding protein, At*: A. thaliana* (marked in red), Bra: *B. rapa*, Bol: *B. oleracea*, Bna: *B. napus*.

**Figure 3:**
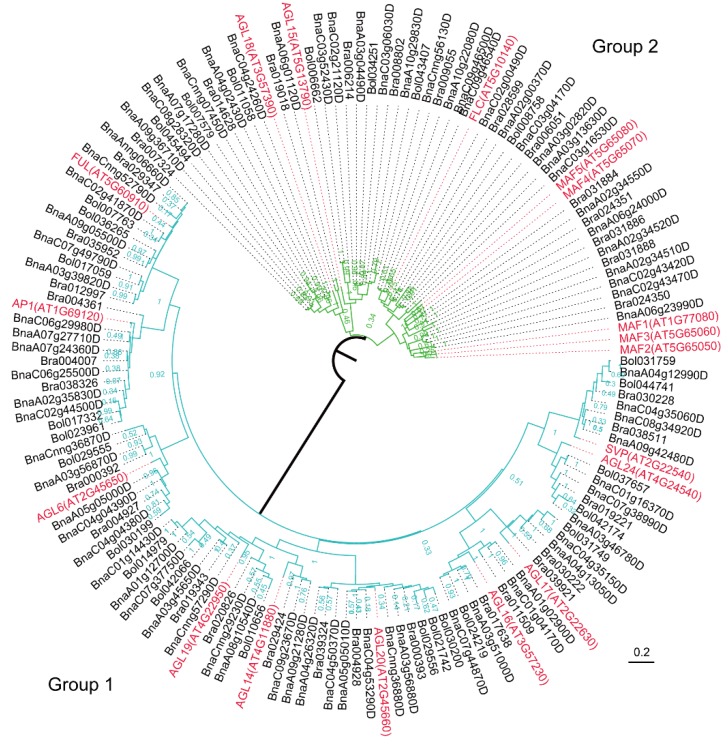
Phylogeny of MADS family proteins in *A. thaliana*, *B. napus*, *B. rapa*, and *B. oleracea*. The phylogenetic tree was constructed using MEGA 7.0 with the neighbor-joining method (1000 bootstrap replicates) and displayed using FigTree v1.4.0. MADS proteins involved in flowering are clustered into two distinct groups (Group 1, the turquoise clade; Group 2, the green clade). The scale bar denotes the number of nucleotide replacements per site. MADS: MCM1, AGAMOUS, DEFICIENS, and SRF, At*: A. thaliana* (marked in red), Bra: *B. rapa*, Bol: *B. oleracea*, Bna: *B. napus*.

Phylogenetic analysis of the PEBP family proteins from *A. thaliana*, *B. napus*, *B. rapa*, and *B. oleracea*, revealed that the PEBP family was clearly divided into two groups: FT and TSF (Group 1), and TFL1, ATC, and BFT (Group 2) (Figure 2). Group 1 contained 13 *Brassica* members and two *A. thaliana* members. Group 2 possessed 29 *Brassica* members and three *A. thaliana* members. All putative sequences for each PEBP subfamily member from the four Brassicaceae species formed a well-supported clade. Orthologs from *A. thaliana*, *B. napus*, *B. rapa*, and *B. oleracea* formed a specific Brassicaceae subfamily clade that was distinct from other. These clades were detected in MADS-box family members as well. The MADS-box family involved in flowering was classified into two large subfamilies (Figure 3). Group 1 contained 10 small subfamilies, including the AGL6, AGL7, AGL8, AGL14, AGL16, AGL17, AGL19, AGL20, AGL22, and AGL24 subfamilies. Group 2 possessed four small subfamilies, including the MAF1-5, FLC, AGL15, and AGL18 subfamilies, and MADS-box proteins involved in various flowering pathways. 

All the MCM1, AGAMOUS, DEFICIENS, and SRF (MADS)-box proteins in Group 2 had a negative effect on flowering time, while most members in Group 1 had a positive effect, with the exception of the SVP (AGL22) and AGL16 subfamilies, which had a negative effect, and the AP1 (AGL7) subfamily, which had no identifiable effect. In Group 2, the MAF subfamily, MAF1-3, clustered together to form the MAF subclade with MAF4-5 (Figure 3), implying that two ancestral copies exist in the MAF subfamily, one for MAF1-3 and another for MAF4-5. Moreover, the FLC subclade displayed a close relationship with the MAF subclade, indicating a common ancestral sequence for this closely related subclade. This evolutionary relationship was displayed in other subclades as well, such as in the AGL6-8, AGL22, and AGL24 subclades. 

### 2.6. Selective Pressure Calculations of Flowering-Time Genes

**Figure 4:**
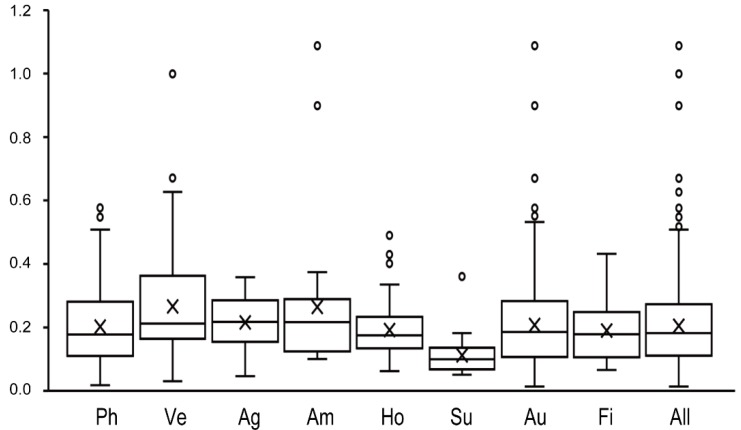
Direction and magnitude of selective pressures acting on different pathways in *B. napus*. Quantile boxplots show the distribution of *Ka/Ks* values for orthologous homologous gene pairs. The horizontal bar in each box indicates the median value. The crossbar in each box indicates the average value. The upper and lower bars correspond to the upper and lower adjacent values outside the inter-quartile range. Ph: photoperiodic, lighting, and signaling pathway; Ve: vernalization pathway; Ag: aging pathway, Am: ambient temperature pathway; Ho: hormone pathway; Su: sugar pathway; Au: autonomous pathway; Fi: flowering-time integrator pathway; All: all flowering-time genes identified in our study.

To determine the selective pressures of flowering-time genes, *Ka*, *Ks*, and *Ka/Ks* were calculated for *B. napus* (see Section 4. Materials and Methods). The *Ka* ranged from 0.0056 (*BnaC02g34760D*) to 2.2319 (*BnaA01g19030D*), with an average of 0.1077 (Table S8), and *Ks* estimates ranged from 0.1859 (*BnaA09g05500D*) to 2.7018 (*BnaC03g16130D*), with a mean of 0.5193 (Table S8). The *Ka/Ks* ratios ranged from 0.0130 (*BnaC02g34760D*) to 1.0874 (*BnaA07g01200D*), with an average of 0.2052 (Figure 4, Table S8), and 43.14% of genes had *Ka/Ks* estimates higher than the average. Only *BnaA07g01200D* had a *Ka/Ks* ratio greater than one, implying that this gene was subjected to positive selection. The average *Ka/Ks* ratio in the A subgenome was only slightly lower than in the C subgenome (Table S8), indicating that a similar evolutionary rate and selection pressure exist for flowering-time genes in both the A and C subgenomes. In addition, the average *Ka*/*Ks* ratios varied between the different flowering-time pathways and were highest in the vernalization pathway (0.2653), followed by those in the ambient temperature pathway (0.2634), while the lowest was in the sugar pathway (0.1120) (Figure 4, Table S8).

The gene with the highest *Ka/Ks* ratio, *BnaC02g34760D* (1.0874), is an ortholog of *AtFVE* (*AT2G19520*) and functions in both the autonomous and ambient temperature pathways. We identified seven orthologs of *AtFVE* in *B. napus*, including *BnaA01g19030D*, *BnaA09g09860D*, *BnaA09g44120D*, *BnaAnng33220D*, *BnaC07g01700D*, *BnaC08g36680D*, and *BnaC09g09900D* (Table S3). Six of these genes had a *Ka/Ks* ratio of less than 0.3, and only *BnaA01g19030D* had a higher *Ka/Ks* ratio (0.8988, Table S8). Moreover, the *Ka/Ks* ratio of *FRL2* (*BnaA03g13320D*, vernalization pathway) was close to one (0.9989, Table S8), indicating that this gene was subject to neutral selection.

In addition, the *Ka/Ks* ratios of flowering-time genes in the vernalization pathway showed higher variation than those in other pathways (Figure 4, Table S8), implying that vernalization-pathway genes were not only subjected to greater selection pressures but also experienced more functional differentiation than genes in other pathways. 

### 2.7. Expression Patterns of Vernalization-Pathway Genes

**Figure 5:**
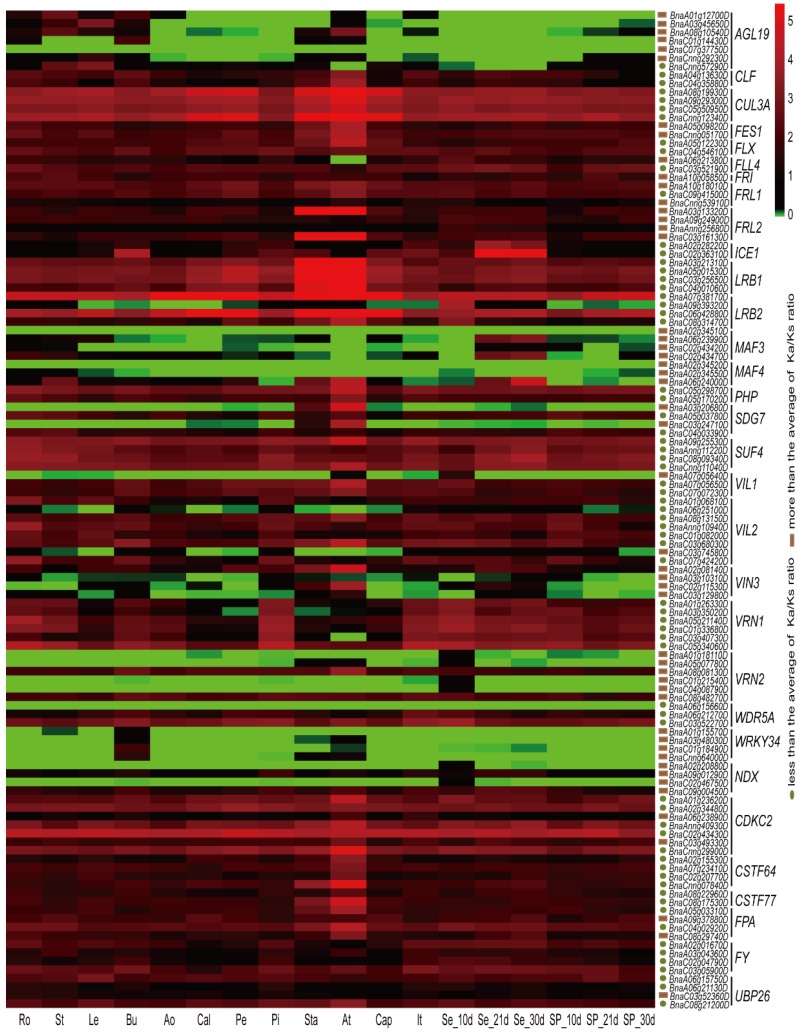
Organ-specific expression profiles of flowering-time genes in the vernalization pathway of *B. napus*. The expression patterns of these genes were obtained from RNA-seq data. The color bar at the upper right side of the figure represents Log_2_ (FPKM + 1), with green representing little or no expression. The root (Ro), stem (St), leaf (Le), bud (Bu), anthocaulus (Ao), calyx (Cal), petal (Pe), pistil (Pi), stamen (Sta), anther (At), capillament (Cap), and inflorescence top (It) organs were harvested at the initial blooming stage, while seeds (Se) and silique pericarps (SP) were harvested after 10, 21, and 30 days in the field. The brown box represents high *Ka/Ks* gene pairs and the dark green ellipse represents low *Ka/Ks* gene pairs. FPKM: fragments per kilobase of exon model per million reads mapped.

To investigate the expression profiles of vernalization-pathway genes, which rapidly evolved in *B. napus*, eight reproductive organs and six vegetative organs were chosen. Although the average expression levels varied among the different vernalization-pathway genes, similar expression patterns were observed among homologs and among the same gene subfamilies (Figure 5). 

Most genes were expressed in flowering organs and tended to be strongly expressed in one or two organs. For example, four *LRB1* members were expressed in all organs investigated, but their expression was mostly in the stamen and anther. High expression in all organs was observed in four *CUL3A* members (*BnaA08g19930D*, *BnaA09g29300D*, *BnaC05g50950D*, and *BnaCnng12340D*), as well as in two *CDKC2* genes (*BnaC02g43430D* and *BnaCnng29900D*) and two *LRB2* (*POB1*) genes (*BnaA07g38170D* and *BnaC06g42880D*) (Figure 5). In addition, strong expression of *BnaA07g38170D* and *BnaC06g42880D* was not only detected in the anther, but also in the anthocaulus, calyx, petal, and capillament. Several vernalization-pathway genes were preferentially expressed in the anther, including two *FRL2*, two *PHP*, two *SUF4,* and four *CDCK2* members. Twenty-nine vernalization-pathway genes had weak or no expression in most organs, especially in the reproductive organs, including eight *AGL19*, four *MAF3*, four *MAF4*, two *SDG7*, four *VRN2*, four *WRKY34*, and one *VIL1*, *NDX*, and *WDR5A* members (Figure 5).

**Figure 6:**
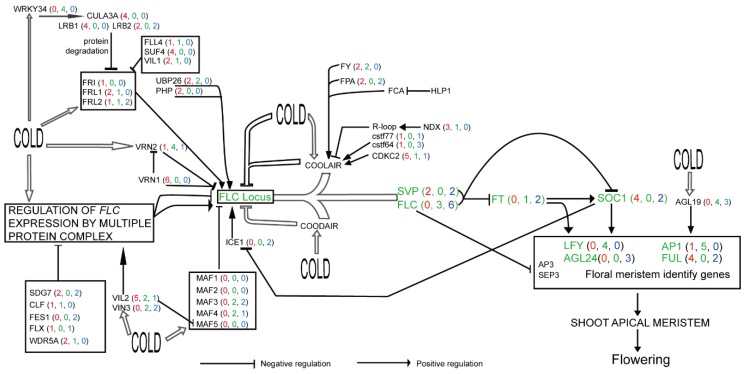
The flowering-time vernalization pathway in *B. napus*. This pathway was modified from a previous model [11]. Genes directly involved in the vernalization pathway in our study are marked in black, while genes belonging to the flowering-time integrator pathway but playing central roles in vernalization are marked in green. Values in parentheses, from left to right, represent the numbers of highly expressed genes (red), weakly expressed genes (green), and organ-specific genes (blue) in the same subfamily. The open arrows represent genes that could be induced by environmental changes and cold treatment.

**Figure 7:**
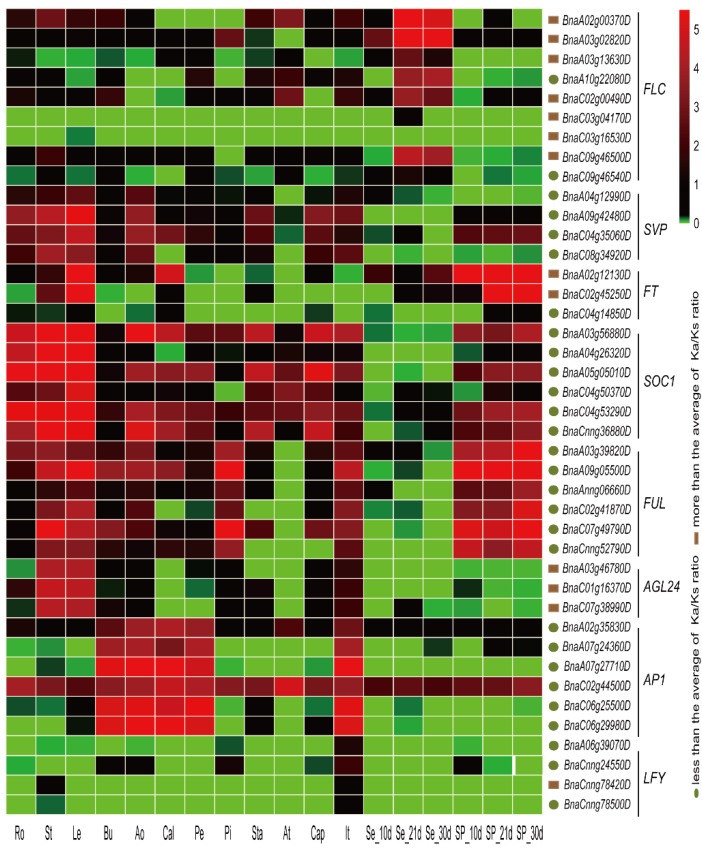
Organ-specific expression profiles of some flowering-time integrator genes that play crucial roles in vernalization in *B. napus*. The expression patterns of these genes were obtained from the RNA-seq data. The color bar at the upper right side of the figure represents Log_2_ (FPKM + 1), with green representing little or no expression. The root (Ro), stem (St), leaf (Le), bud (Bu), anthocaulus (Ao), calyx (Cal), petal (Pe), pistil (Pi), stamen (Sta), anther (At), capillament (Cap), and inflorescence top (It) organs were harvested at the initial blooming stage, while seeds (Se) and silique pericarps (SP) were harvested after 10, 21, and 30 days in the field. The brown box represents high *Ka/Ks* gene pairs and the dark green ellipse represents low *Ka/Ks* gene pairs. FPKM: fragments per kilobase of exon model per million reads mapped.

Since some flowering-time integrator genes play key roles in vernalization, such as *FLC*, *SVP*, *FT*, *SOC1*, *FUL*, *AGL24*, *AP1,* and *LFY* (Figure 6), their expression profiles were also assessed in this study. These flowering-time integrator genes displayed diverse expression patterns in different subfamilies (Figure 7). Five *FLC* genes were highly expressed in seeds at 21 and 30 days after flowering, especially *BnaA03g02820D* and *BnaA02g00370D*, but other *FLC* members were weakly or not expressed in most organs. Three *SVP* and three *AGL24* genes were highly expressed in leaves and stems. Two *FT* members were expressed at extremely high levels in leaves and silique pericarps during the developmental stages studied. All *SOC1* family members were strongly expressed in at least one organ, including flowering organs (Figure 7). *FUL* family members displayed high expression levels in leaves, stems, and silique pericarps at 10, 21, and 30 days after flowering, and *BnaA09g05500D* and *BnaC07g49790D* showed high expression levels in flowering organs. Unlike the subfamilies mentioned previously, six *AP1* subfamily members were, for the most part, expressed specifically in five flowering organs (bud, anthocaulus, calyx, petal, and inflorescence top), *BnaC02g44500D* was expressed in all organs examined. However, *LFY* subfamily members were expressed at extremely low levels or not expressed in all organs examined (Figure 7). By comparing gene expression patterns between the vernalization pathway and flowering-time integrator pathways, we showed that genes in the vernalization pathway have a wider breadth of expression (number of organs in which the gene is expressed) than genes in the flowering-time integrator pathway. In this pathway, expression was observed mostly in specific organs (Figure 5 and Figure 7). In addition, genes in the flowering-time integrator pathway were more likely to be strongly expressed in non-flowering organs (except for members of the *AP1* subfamily, which highly expressed in flowering organs), illustrating their multiple functions in the control of flowering.

### 2.8. The relationship between Ka/Ks Ratios and Expression Patterns

**Figure 8:**
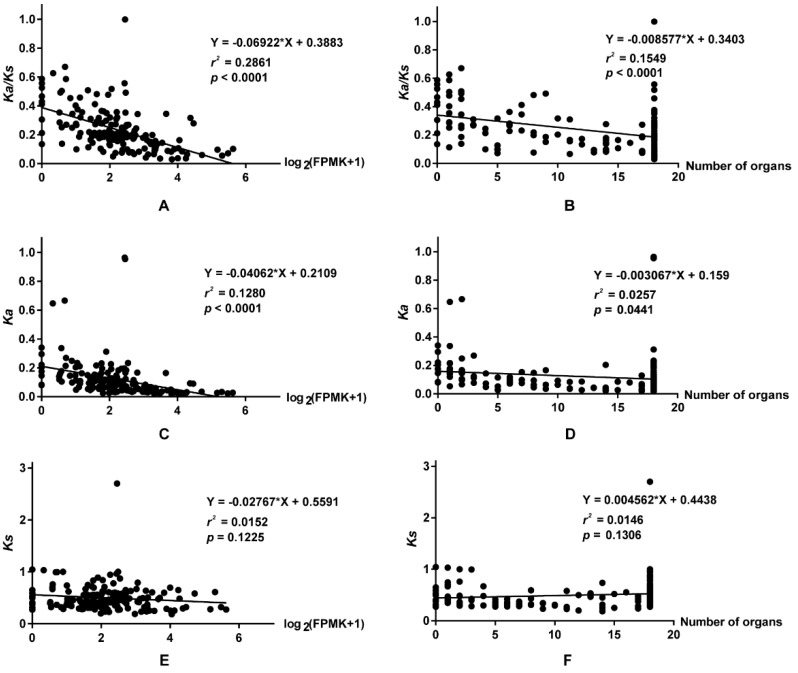
Correlation analyses between *Ka/Ks*, *Ka*, and *Ks* with average expression levels and expression breadth in *B. napus*. The average expression levels were calculated using the sum of the expression levels in organs divided by the numbers of organs. Here, the organs were defined as the organs with expression levels higher than 0.5. (**A**,**C**,**E**) represent the correlation between *Ka/Ks*, *Ka*, and *Ks* with expression levels, while (**B**,**D**,**F**) represent the correlation between *Ka/Ks*, *Ka*, and *Ks* with expression breadth, respectively. The expression data were normalized with Log_2_ (FPKM + 1). FPKM: fragments per kilobase of exon model per million reads mapped.

To investigate the relationship between selective pressure and expression patterns, the correlation between *Ka/Ks* ratios and expression breadth, and between *Ka/Ks* ratios and average expression levels, was calculated. The correlation coefficient (*r*^2^) between the *Ka/Ks* ratios and the average expression levels was 0.2861 (*p* < 0.0001) (Figure 8A), while that between the *Ka/Ks* ratios and expression breadth was 0.1549 (*p* < 0.0001) (Figure 8B), indicating that selective pressure was negatively correlated with the expression levels and organs. In the *B. napus* vernalization pathway, genes undergoing rapid evolution displayed low, organ-specific expression. For example, of the six *AGL19* members, *BnaA01g12700D* (*Ka/Ks* = 0.3168) was primarily expressed in the stem, *BnaA08g10540D* (*Ka/Ks* = 0.3628) was primarily expressed in the anther, and *BnaA03g45650D* (*Ka/Ks* = 0.2729) was highly expressed in the leaves (Figure 5, Table S8). However, *BnaC01g14430D* (*Ka/Ks* = 0.3099), *BnaC07g37750D* (*Ka/Ks* = 0.5570), and *BnaCnng29230D* (*Ka/Ks* = 0.3453) were weakly expressed in all organs, or not expressed at all. By contrast, genes with low *Ka/Ks* ratios, such as *CUL3A*, *LRB1*, *LRB2,* and *SUF4,* were constitutively expressed in various organs (Figure 5). This phenomenon was more pronounced in homologous subfamilies (Figure 5 and Figure 7). For example, in the *SDG7* subfamily, *BnaA03g20680D* (*Ka/Ks* = 0.3454) and *BnaC03g24710D* (*Ka/Ks* = 0.3436) were highly expressed in the anther, whereas *BnaA05g03780D* (*Ka/Ks* = 0.1837) and *BnaC04g03390D* (*Ka/Ks* = 0.1849) were expressed in most organs.

The correlation coefficient calculations revealed that *Ka* was also negatively correlated with average expression levels and expression breadth (Figure 8C,D), while no relationship was detected between *Ks* and the expression patterns of the vernalization-pathway genes.

## 3. Discussion

Flowering-time genes have been studied widely in numerous crops, and several genes involved in flowering time, such as *FLC*, *CO*, *FT*, and *FRI*, have been identified in *Brassica* crops [26,28,29,38,39]. In this study, a total of 1064, 510, and 524 flowering-time genes were identified from *B. napus*, *B. rapa*, and *B. oleracea*, respectively. Compared to other crops or plants, it appears that *Brassica* crops possess more flowering-time genes [22,24,40], due to whole genome triplication (WGT) and a genome merging event during their evolution [32]. However, no orthologs for some flowering-time genes were found in the *Brassica* species we studied, suggesting gene loss or genome contraction may have occurred in *Brassica* after WGT [32,34,41]. As *Brassica* crops nonetheless experience different endogenously and externally factors to facilitate flowering, it is assumed that these lost genes do not impact the central flowering processes [24], or that *Brassica* evolved other genes to replace their functions. Genes identified using HMMER and BLASTP analyses exhibited the most sequence similarity with their orthologs in *A. thaliana*, as well as a higher degree of sequence conservation within the protein functional domains [18], but genes identified using a reciprocal BLASTP analysis displayed relatively lower sequence similarity with their *Arabidopsis* orthologs. This illustrates that functional domains play crucial roles in the prediction or identification of flowering ortholog proteins in crop species [42].

The number of flowering-time integrator pathway genes in *Brassica* species identified in this study was different from previous studies. Two copies of *FT*, four of *FLC* and three of *CO* had been described in *B. rapa* previously [28,29], but only one *FT*, three *FLC,* and one *CO* copies were identified from *B. rapa* in our study. A similar phenomenon was observed in *B. oleracea*, in which we identified two copies of *FLC*, one of *CO* and one of *FT*, whereas four, five and three members have been described previously [39,43,44]. In *B. napus*, five copies of *FLC* were identified previously [45], while nine copies were identified using our methods. Due to no *Brassica* reference genomes, the numbers of flowering-time genes identified in previous studies were often inaccurate. To compare the differences between previous studies and this study, we selected the FLC protein sequences (identified in previous studies and this study) as an example. Sequences of three BnaFLC proteins (BnaA02g00370D, BnaA03g13630D and BnaC02g00490D) identified in this study were the same with those in earlier studies (Figure S4), while the rest six BnaFLC proteins distributed on different chromosomes all showed high similarity and contained the SRF-TF/K-box domains, suggesting that fewer FLC members identified in previous studies may be caused by the complexity of the *Brassica* genomes. We found that the *B. rapa* FLC proteins identified in earlier showed sequence lost at the N terminal sequences (Figure S5). Sequence difference of *B. oleracea* FLC proteins (Figure S6) might be also caused by the genome complexity. Recently studies identified a plenty of flowering-time genes in *Brassica* using WGAS (genome-wide association study) and transcriptome analysis [46,47]. Comparison of selected subfamilies revealed that flowering-time genes identified in our study were more than those in other researches, and most of the genes identified in previous studies could be found in our study (Table S9). The rest genes identified in previous studies but not found in our study might be orthologs of other genes in *A. thaliana*, since the functional domains were different (Tables S3, S9 and S10), suggesting that our gene identification results were more reliable than previous studies. The expression profiles of flowering-time genes in different organs have been analyzed and investigated in several species [21,22,24,40,43,48]. In *A. thaliana*, the expression of flowering-time genes was detected in at least one of the 63 organs and development stages analyzed [42]. In barley, 91% of the flowering-time genes analyzed were expressed in at least one of 15 organs and 75% of the flowering-time genes analyzed in wheat were expressed in at least one of 13 organs [42]. In our study of *B. napus*, approximately 63% of the flowering-time genes involved in vernalization were expressed in at least 10 organs, which is relatively low compared to previous studies such as Peng et al. [42]. This is most likely due to the organ-specific expression of some of the genes involved in vernalization (Figure 5 and Figure 7). In *Raphanus sativus*, 183 (from 254) flowering-time genes accumulated in flowering organs and genes from seven subfamilies presented preferential or specific expression patterns in flowers [24]. In our study, all the expressed genes showed expression in at least one flowering organ, and many genes from subfamilies were preferentially expressed in the anther.

The expression profiles of flowering-time genes were remarkably different in the pre-anthesis anthers of wheat and barley, and in the pollen of *A. thaliana*, compared to the other organs [42], suggesting that the accumulation of their transcripts may prolong anther (pollen) activity, increasing the probability of cross-fertilization. In our study, the expression of eight flowering-time genes, *BnaA06g15660D* (*MAF3*), *BnaA02g34520D* (*MAF4*), *BnaA02g34510D* (*VRN2*), *BnaC07g37750D* (*WRKY34*), *BnaC03g04170D* (*FLC*), *BnaC03g16530D* (*FLC*), *BnaA04g12990D* (*FLC*), and *BnaC04g14850D* (*FT*), was not detected in any organ, but the corresponding homologs in the same subfamily showed weak expression patterns in most organs or were highly expressed in specific organs. This is similar to the expression patterns of flowering-time genes in cotton [22], implying that non-functionalization or sub-functionalization occur in certain flowering-time gene subfamilies. However, in cotton, no expression of *CUL1* was detected in any organ [22], but in our study, its ortholog in *B. napus*, *CUL3A* (*BnaA08g19930D*, *BnaA09g29300D*, *BnaC05g50950D*, and *BnaCnng12340D*), showed strong expression in all organs. This suggests that its functions were pseudogenized or repressed in cotton.

The *FRI* gene in *A. thaliana* was shown be expressed ubiquitously and its expression levels in leaves and flowers were very similar [49]. Our expression analysis confirmed that result, but we found that *FRI* was not expressed at high levels in any organ or at any developmental stage in *B. napus*. Its homologs *FRL1* and *FRL2* showed high expression in most organs and in anthers, respectively, implying that functional substitutions among homologs may occur in vernalization pathways in *Brassica*. Strangely, although *FLC*, *FT*, and *SVP* are crucial genes in vernalization, members from the former two subfamilies did not show strong expression profiles in flowering organs in our study, perhaps due to the intermodulation of genes (Figure 6). Two floral meristem identity subfamily genes were highly expressed in most flowering organs, indicating that they control *B. napus* flowering time through their accumulation via the process of vernalization. In addition, most of the remaining vernalization genes displayed expression patterns and organ-specific expression similar to those presented in previous studies such as Wang et al. [24].

Many gene duplications are the result of the WGT event, and subsequent functional differentiation of genes then occurred during evolution. Based on the models of gene duplication, duplicated genes (paralogs) may undergo non-functionalization (loss of function), neofunctionalization (gain of novel function), or sub-functionalization (separation of original function) [50,51,52,53]. This functional variation is generally seen as a response to selective pressures.

In this study, gene functional differentiation was detected in several duplicated genes. In the vernalization pathway, 35 (22.15%) genes have experienced or are undergoing non-functionalization and more than 50% of genes experienced neofunctionalization or sub-functionalization. For example, *AGL19* in *B. napus* contains seven members and one member (*BnaCo7g37750*) has become silent due to non-functionalization, while the rest of the members display varied expression patterns. This implies that their biological function changed through neofunctionalization or sub-functionalization during the process of evolution. Moreover, the conserved functions are retained in the duplicated genes, because of their highly similar expression patterns in all organs analyzed, including the *CUL3A*, *LRB1*, and *SUF4* subfamilies. This illustrates the functional diversity of flowering-time genes in *B. napus*, as well as the different evolutionary rates of duplicated genes (paralogs).

Previous studies revealed that evolutionary rates are often strongly correlated with gene expression, including both expression level [54,55,56] and expression breadth [57,58]. In *Brassica*, previous research has shown that *Ka/Ks* ratios are negatively correlated with expression level and positively correlated with organ specificity [59]. Our expression analysis of flowering-time genes involved in vernalization supports these results. The *Ka* for highly expressed genes was significantly lower than for organ-specific genes or for those with weak expression, within similar synonymous substitutions. This illustrates that non-synonymous substitutions are the major determinants of the levels and breadth of expression [57,58,60] and the main evolutionary forces in the rapid evolution of the vernalization genes of *B. napus*. This is understandable because most mutations in non-synonymous sites are usually considered to be deleterious to individuals, with the result that most are soon lost from the population [61], leading to a further reduction of the *Ka* value and *Ka/Ks* ratio. This phenomenon also explains why most genes evolved under purifying/negative selection, regardless of the genome to which they belong [59]. However, non-synonymous substitutions are often accompanied by natural environmental pressures during evolution, and therefore cause genes to lose their function or gain a novel function. In summary, non-synonymous substitutions are the best mode for the rapid evolution of genes and provide a basic theory for the alteration, retention, and loss of gene function in plants. These findings also lay a foundation for further understand the relationship between gene expression patterns and their evolutionary selection pressures in plants.

## 4. Materials and Methods

### 4.1. Data Resources

Proteomic, genomic, and coding sequences of *A thaliana* were obtained from the *Arabidopsis* Information Resource (TAIR, http://www.arabidopsis.org), and those of *Brassica napus* were acquired from the *B. napus* database (http://www.genoscope.cns.fr/brassicanapus/). Those of *B. rapa* and *B. oleracea* were downloaded from the *Brassica* Database (BRAD, http://brassicadb.org/brad). A total of 295 flowering-time coding genes from *A. thaliana* were retrieved from the FLOR-ID (http://www.flor-id.org).

### 4.2. Identification of Flowering-Time Genes in Brassica species

Protein sequences of flowering-time genes from *A. thaliana* were imported firstly into PfamScan (http://www.ebi.ac.uk/Tools/pfa/pfamscan/) to identify functional domains and to obtain their Pfam classifications with an E-value cut-off of 1e–5. The flowering-time genes were then divided into two groups: domain-containing genes and non-domain-containing genes.

For domain-containing genes, their Hidden Markov Model (HMM) profiles were acquired from the HMMER web server (https://www.ebi.ac.uk/Tools/hmmer/) and used as queries to search against all the *Brassica* protein sequences using the HMMER V3.0 program (Ashburn, VA, USA) (version 3.1b2, [62]) to identify candidate genes containing the corresponding functional domains, with an E-value of more than the default “inclusion threshold” as the threshold. To accurately identify flowering-time domain-containing genes in the three *Brassica* species, all the filtered candidate genes were then used as queries using the Basic Local Alignment Search Tool Protein (BLASTP) program (Bethesda, MD, USA) ([63]) to search against the *A. thaliana* proteome database to investigate their corresponding orthologs, with a threshold E-value of 1e-5 and a minimum alignment coverage of 50% [64].

For non-domain-containing flowering-time genes in *A. thaliana*, their protein sequences were used as queries in a reciprocal BLASTP analysis to search against the *Brassica* proteome database developed in our study, at the threshold and minimum alignment coverage parameters described as above.

### 4.3. Chromosomal Location and Gene Expansion Analysis

To identify the chromosomal location of each flowering-time gene, all the gene sequences in the three *Brassica* species were used in a BLASTN analysis against their corresponding genome sequences. The best BLASTN hit for each gene was used to obtain its chromosomal distribution. MapChart 2.0 (Wageningen, The Netherlands) [65] was used to draw graphical representations of their physical position on the corresponding chromosomes in *Brassica* species.

To compare the expansion status in different flowering-time pathways among *B. napus*, *B. oleracea*, and *B. rapa*, the following formula was used to calculate the gene number ratio (*Γ*) in each pathway.

*Γ* = (C1/C2)/(N1/N2)

C1: gene number in flowering-time pathway *A* in *B. rapa* or *B. oleracea*
C2: gene number in flowering-time pathway *A* in *B. napus*
N1: total coding gene number in *B. rapa* or *B. oleracea*
N2: total coding gene number in *B. napus*

The C1/C2 ratio represents the ratio of genes in flowering-time pathway *A* in *B. rapa* or *B. oleracea* to their orthologs in *B. napus* and the N1/N2 ratio indicates the ratio of coding genes in *B. rapa* or *B. oleracea* to those in *B. napus*. If *Γ* is less than one, the flowering-time pathway *A* in *B. napus* experienced an expansion event, while if greater than one, a contraction event occurred in this pathway.

### 4.4. Phylogenetic Analysis

To understand the evolutionary relationships among flowering-time gene families in the three *Brassica* species, the PEBP and MADS-box protein families in *B. napus*, *B. rapa*, *B. oleracea*, and *A. thaliana* were selected as representatives to generate phylogenetic trees. Multiple sequence alignments of proteins were performed by ClustalW2 software (Dublin, Ireland) [66] with default parameters.

Three methods, neighbor-joining (NJ), maximum likelihood (ML), and Bayesian (BI), were used to unravel the phylogenetic relationship of the target gene families. NJ trees were performed using a Molecular Evolutionary Genetics Analysis (MEGA) 7.0 (Tokyo Metropolitan University, Tokyo, Japan) [67] based on a *p*-distance model of uniform rate substitutions. To ensure the accuracy of NJ trees, a non-parametric bootstrap method was subjected to bootstrap replication of 1000. The model used to construct ML and BI trees was determined using Model Generator v0.85 (http://mcinerneylab.com/software/modelgenerator/, Tables S11 and S12). The ML trees were constructed using the online PhyML server (PhyML 3.0, http://www.atgc-montpellier.fr/phyml/, [68,69]) with the JTT + G (PEBP family) and JTT + I + G (MADS-box family) substitution models. The following parameters were used to infer evolutionary relationships among the sequences: model of rate heterogeneity: Discrete Gamma (PEBP family) and Discrete Gamma + Invariable sites (MADS-box family); number of rate categories: 4 (PEBP family) and 5 (MADS-box family); gamma distribution parameter *α*: 0.69 (PEBP family) and 1.63 (MADS-box family), and 100 bootstrap replicates (Figure S7). BI trees were constructed using MrBayes 3.2.6 (http://mrbayes.sourceforge.net) [70,71]. To generate the BI tree, the JTT + G (PEBP family) and JTT + I + G (MADS-box family) substitution models were selected and run for 5,000,000 generations, printing every 100,000 generations and sampling every 1000 generations (nchains = 4, temp = 0.2) for these two gene families (Figure S8). All phylogenetic trees were visualized using FigTree v1.4.2 (http://tree.bio.ed.ac.uk/software/figtree/).

To identify the best topology of phylogenetic trees, the corresponding NJ, ML, and BI trees were analyzed using CONSEL (http://stat.sys.i.kyoto-u.ac.jp/prog/consel/, [72]). By comparing their corresponding topologies, the NJ tree with the best topology was chosen for further evolutionary analyses for both the PEBP and MADS-box protein families (Table S13).

### 4.5. Ka/Ks Calculation of Flowering-Time Genes in B. napus

The non-synonymous (*Ka*)/synonymous substitution (*Ks*) ratio of orthologous gene pairs reflects the evolutionary selection patterns of the corresponding genome. In the calculation of *Ka*, *Ks,* and *Ka/Ks*, the coding sequences (CDSs) of flowering-time genes in *B. napus* and *A. thaliana* firstly underwent pairwise alignments using MUSCLE (http://www.ebi.ac.uk/Tools/ msa/muscle/, [73]) with codon alignment. Then, the aligned sequences were used as input files integrated into the KaKs_Calculator2 software (Tempe, AZ, USA) [74] to compute *Ka*, *Ks,* and *Ka/Ks* values using the Li-Wu-Luo model [75]. The *Ka/Ks* ratios less than one, equal to one, and greater than one was considered to represent negative, neutral, and positive selection, respectively.

### 4.6. Expression Profiles of Vernalization-Pathway Genes in B. napus

To investigate the spatio-temporal expression patterns of vernalization-pathway genes, publicly available RNA-seq data (BioProject ID PRJNA358784) from different organs in *B. napus* cultivar ‘ZS11’ were used to quantify gene expression levels, based on their FPKM values using Cufflinks [76] with default parameters. For each sample, two independent biological replicates were included. The expression values of the vernalization-pathway genes were retrieved from the RNA-seq results, which include 14 different organs, root (Ro), stem (St), leaf (Le), bud (Bu), anthocaulus (Ao), calyx (Cal), petal (Pe), pistil (Pi), stamen (Sta), anther (At), capillament (Cap), inflorescence tip (It), seeds (Se), and silique pericarps (SP), harvested at 10, 21, and 30 days after flowering, and then normalized by Log_2_ (FPKM + 1).

### 4.7. Statistical Analysis

To determine the coefficient of correlation between the selective pressure and expression pattern, the average expression level of a gene was calculated using the sum of the expression levels in the organs divided by the numbers of organs, using organs with expression levels higher than 0.5. The expression breadth was the number of organs in which the genes are expressed and with levels higher than 0.5.

## Figures and Tables

**Table 1:** The flowering-time genes identified in *A. thaliana, B. rapa, B. oleracea,* and *B. napus.*

Pathways	*A. thaliana*	*B. oleracea*		*B. rapa*		*B. napus*	
		Number of Genes	Ratio *	Number of Genes	Ratio *	Number of Genes	Ratio *
Ph	90 (113)	156 (193)	1.71	153 (187)	1.66	318 (390)	3.45
Ve	26 (34)	41 (57)	1.68	46 (61)	1.79	88 (117)	3.44
Ag	2 (8)	4 (13)	1.63	4 (12)	1.50	9 (25)	3.13
Am	5 (10)	6 (12)	1.20	7 (13)	1.30	16 (27)	2.70
Ho	25 (25)	47 (47)	1.88	43 (43)	1.72	99 (99)	3.96
Su	9 (9)	17 (17)	1.89	15 (15)	1.67	29 (29)	3.22
Au	88 (113)	163 (209)	1.85	155 (198)	1.75	325 (413)	3.66
Fi	16 (16)	33 (33)	2.06	34 (34)	2.13	72 (72)	4.50
Ph/Ag	6	9	-	8	-	16	-
Am/Au	2	4	-	4	-	9	-
Am/Ph	2	2	-	2	-	4	-
Am/Ve	1	0	-	0	-	0	-
Ph/Au	15	26	-	24	-	50	-
Ve/Au	8	16	-	15	-	29	-
Total	295	524	1.78	510	1.729	1064	3.607

* Ratio of the number of genes in the Brassica species to the number of *A. thaliana* genes in the equivalent pathway. Note: Numbers in parentheses are the total number of genes involved in the corresponding flowering pathway. For example, 113 in the Ph pathway of *A. thaliana* is equal to 90 (Ph) plus 6 (Ph/Ag) plus 2 (Am/Ph) plus 15 (Ph/Au). Ph: photoperiodic, lighting, and signaling pathway; Ve: vernalization pathway; Ag: aging pathway, Am: ambient temperature pathway; Ho: hormone pathway; Su: sugar pathway; Au: autonomous pathway; Fi: flowering-time integrator pathway.

## Supplementary Materials

Supplementary materials can be found at http://www.mdpi.com/1422-0067/19/11/3632/s1.
