# Supplementary material for: Genome-Wide Identification of Flowering-Time Genes in Brassica Species and Reveals a Correlation between Selective Pressure and Expression Patterns of Vernalization-Pathway Genes in Brassica napus
Source: Int J Mol Sci. 2018 Nov 18;19(11):3632. doi: 10.3390/ijms19113632 (PMC6274771; doi:10.3390/ijms19113632)
Supplement: Supplementary file 1 [file ijms-19-03632-s001.zip › Supplementary (figure).docx]

**Supplementary Material**


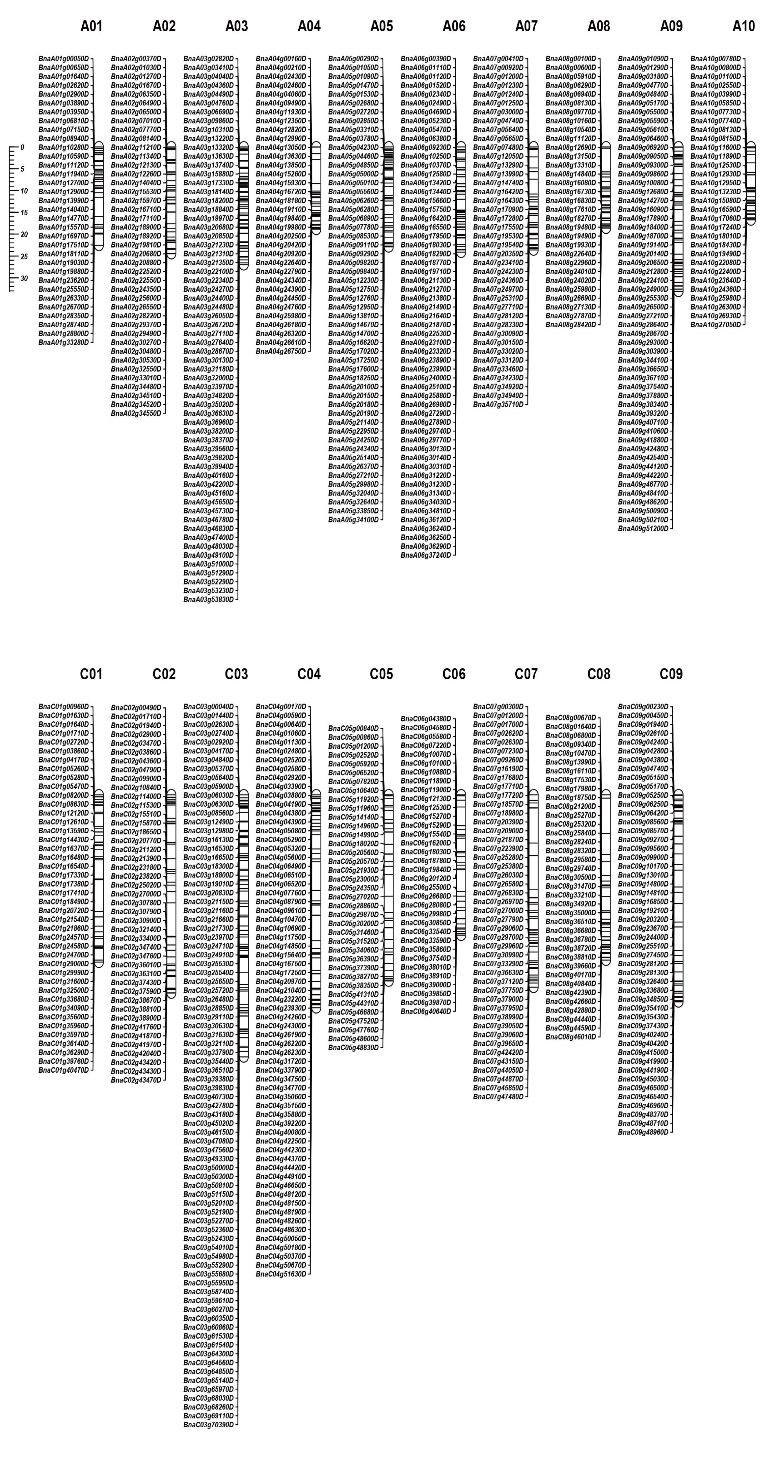


**Figure S1.** Distribution of flowering-time genes on *B. napus* chromosomes. The black line on olive bars indicates the location of flowering-time genes on pseudo-chromosomes. Values corresponding to the scales on the black vertical line indicate physical distance.


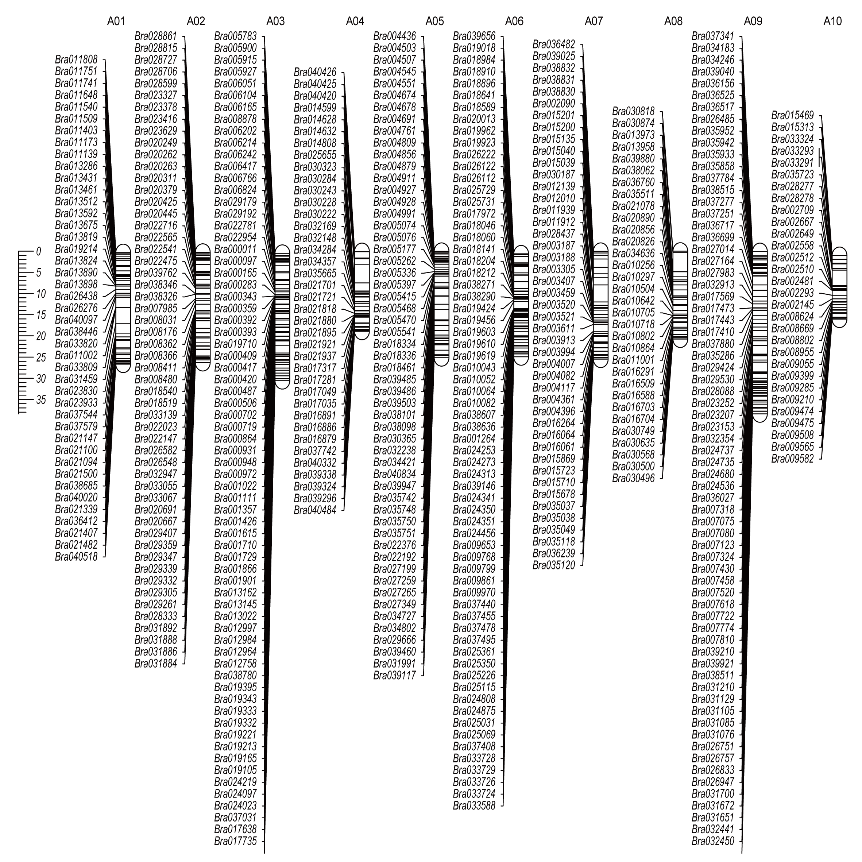


**Figure S2.** Distribution of flowering-time genes on *B. rapa* chromosomes. The black line on olive bars indicates the location of flowering-time genes on pseudo-chromosomes. Values corresponding to the scales on the black vertical line indicate physical distance.


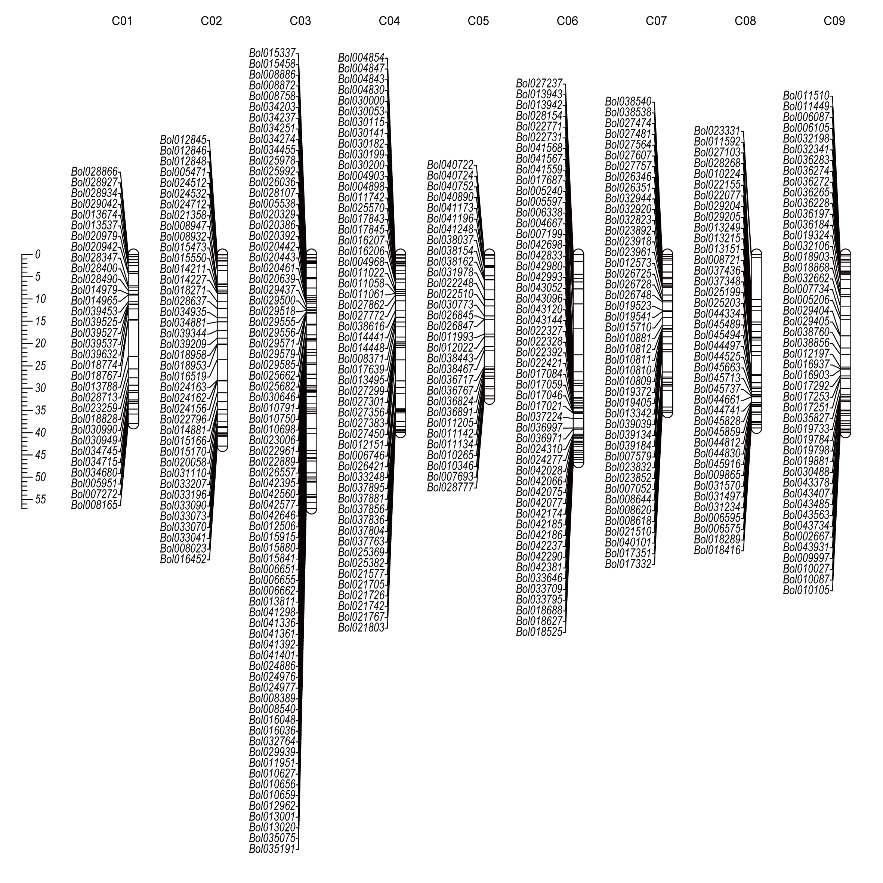


**Figure S3.** Distribution of flowering-time genes on *B. oleracea* chromosomes. The black line on olive bars indicates the location of flowering-time genes on pseudo-chromosomes. Values corresponding to the scales on the black vertical line indicate physical distance.


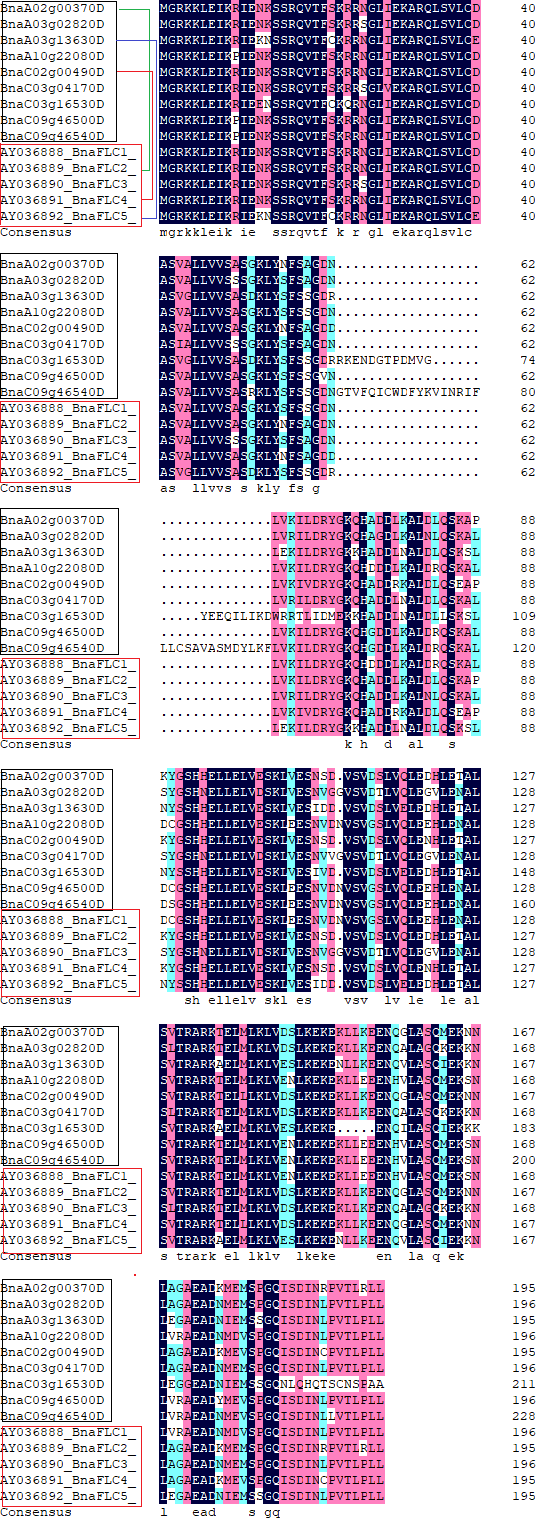


**Figure S4.** Comparison of *FLC* genes in *B. napus* between previously studies and this study. Genes in black box were identified in this study and genes in red box were identified in previously studies. The lines showed that these genes have the same sequences.


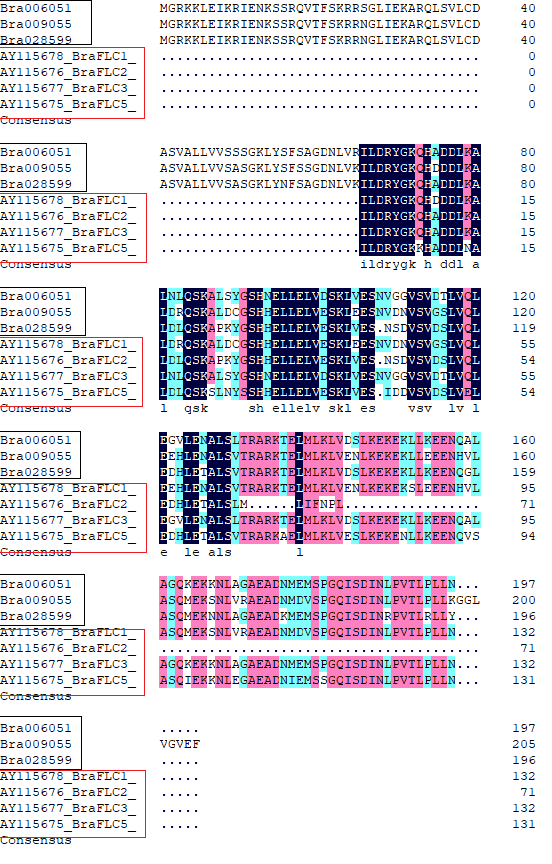


**Figure S5.** Comparison of *FLC* genes in *B. rapa* between previously studies and this study. Genes in black box were identified in this study and genes in red box were identified in previously studies.


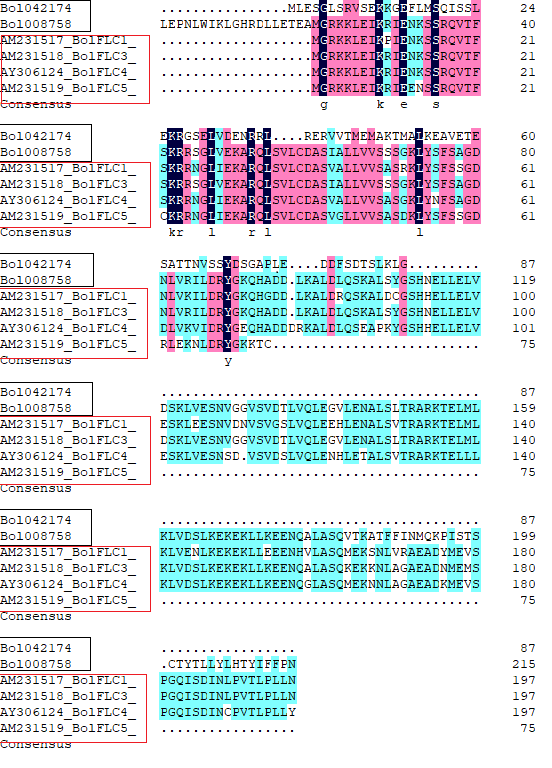


**Figure S6.** Comparison of *FLC* genes in *B. oleracea* between previously studies and this study. Genes in black box were identified in this study and genes in red box were identified in previously studies.


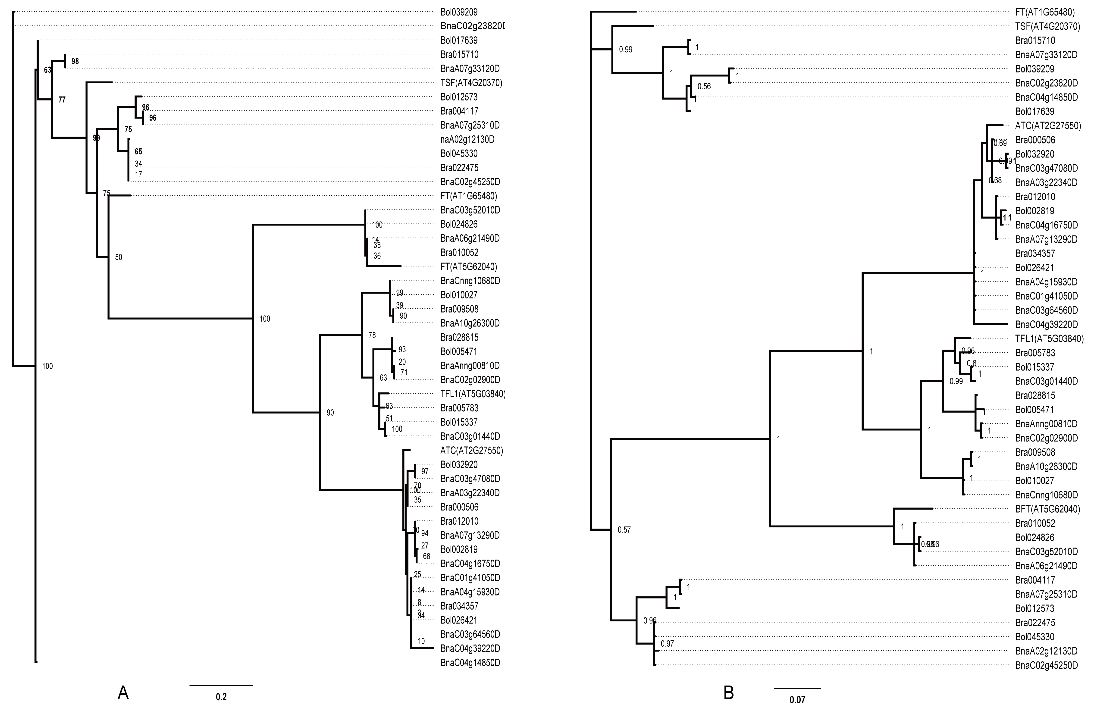


**Figure S7.** Phylogenetic trees of the *PEBP* gene family involved in flowering time in *A. thaliana*, *B. napus*, *B. oleracea,* and *B. rapa*. Amino acid sequences were aligned using MUSCLE. The ML (A) and BI (B) trees were constructed by online PHYLIP with bootstrap analysis (100 replicates) and MrBayes3.2.6. All the trees are displayed using FigTree v1.4.0.


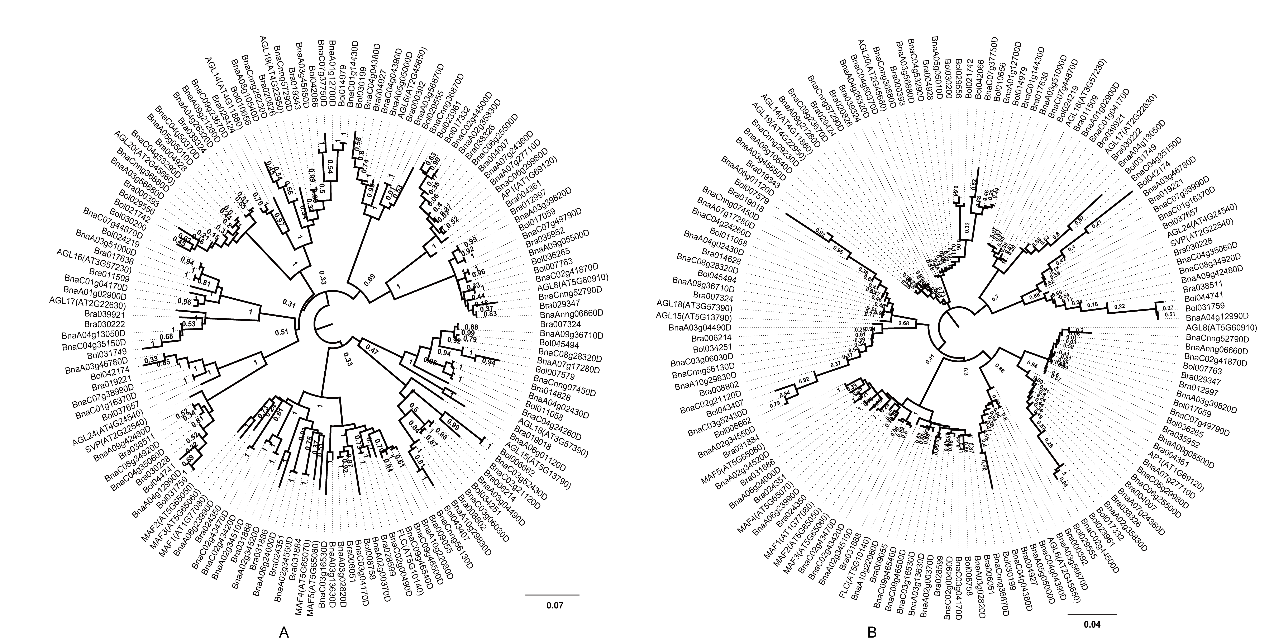


**Figure S8.** Phylogenetic trees of the *MADS* gene family involved in flowering time in *A. thaliana*, *B. napus*, *B. oleracea,* and *B. rapa*. Protein sequences were aligned using MUSCLE. The ML (A) and BI (B) trees were constructed by online PHYLIP with bootstrap analysis (100 replicates) and MrBayes3.2.6. All the trees are displayed using FigTree v1.4.0.

**Table S1** Flowering-time genes described in FLOR-ID.

**Table S2** Domains of flowering-time genes analyzed in *A. thaliana*.

**Table S3** Flowering-time genes identified in *B. napus*.

**Table S4** Flowering-time genes identified in *B. rapa*.

**Table S5** Flowering-time genes identified in *B. oleracea*.

**Table S6** Flowering-time genes not identified in this study.

**Table S7** Distribution of flowering-time genes on *Brassica* species chromosomes.

**Table S8** The *Ka/Ks* values of homologous flowering-time genes between *B. napus* and *A. thaliana*.

**Table S9** Selected flowering time genes identified between recently studies and ours.

**Table S10** Genes not identified in our study but identified by other methods.

**Table S11** Model-fit Ranking according to all measures for the *PEBP* gene family.

**Table S12** Model-fit Ranking according to all measures for the *MADS* gene family.

**Table S13** Comparison of the topologies of the NJ, ML, and BI trees.
